# Editorial: The future food analysis

**DOI:** 10.3389/fnut.2022.1014322

**Published:** 2022-09-23

**Authors:** Tao Pan, Jun-Li Xu, Quancai Sun

**Affiliations:** ^1^Department of Optoelectronic Engineering, Jinan University, Guangzhou, China; ^2^School of Biosystems and Food Engineering, University College Dublin, Dublin, Ireland; ^3^Department of Food Science and Technology, National University of Singapore, Singapore, Singapore

**Keywords:** food analysis, near-infrared spectroscopy, terahertz spectroscopy, spectral imaging, bioactive characterization, process analytical technology

In the field of nutrition science, food analysis is an essential tool for guaranteeing the safety, quality, traceability, and nutritional value of foods in order to comply with legislation and meet rapidly changing consumer demands. With the technical progress of analytical instruments and methods, especially the advanced development of artificial intelligence and deep learning algorithms, food analysis has undergone substantial improvements in accuracy, precision, detection limits, and sample throughput in recent years, thereby expanding its range of practical applications.

This Research Topic brought together important research contributions applying advanced and emerging analytical approaches, the results of which have demonstrated tremendous possibilities for speeding up the resolution of food safety issues; preventing food fraud for authentication, certification, and traceability purposes; and better understanding the bioactivity of food and food ingredients at the molecular level. As these approaches demonstrate, novel analytical methods are starting to replace conventional techniques, many of which are time-consuming, labor-intensive, and less accurate.

For instance, Li et al. proposed a classification framework combining multispectral imaging (MSI) technology with machine learning tools for rapid grading of beef products. They studied a total of 555 beef cut samples (comprising 200 sirloin, 195 shank, and 160 flank samples), of which 445 were used as the training set to build the model while the remaining 110 samples were used as the test set to evaluate the model's performance. Through the use of feature extraction and fusion strategy, multispectral images were converted into single-modal and multiple-modal feature sets and further used as input for machine learning–based classifiers. The results demonstrated that the multiple-modal fusion feature set outperformed the single-modal feature set, and the optimized linear discriminant analysis (LDA) classifier using multiple-modal feature fusion achieved a prediction accuracy rate of over 90%. The MSI-based classification framework is fast and non-destructive, and is capable of providing accurate results in real time. It is therefore suitable for use in contemporary food industry settings as a tool for avoiding fraud and enhancing quality management.

Terahertz (THz) spectroscopy is a promising novel analytical method that is less likely to cause damage to biomolecules, both because the THz waves have relatively low-photon energy and because it is not easily influenced by scattering and fluorescent substances. Using THz spectroscopy, Yang et al. innovatively introduced machine learning methods, such as convolutional neural networks (CNN), to classify coffee bean samples based on their different geographical origins. Principal component analysis (PCA) and genetic algorithm (GA) were applied to wavelength screening, leading to a smaller feature set that best described the difference between origins. They showed that the CNN-based method has a strong predictive ability, with an accuracy rate of 90%. THz spectroscopy combined with the machine learning method significantly enhanced the accuracy of coffee bean origin classification, demonstrating that it has strong application potential in the classification and identification of agricultural products.

Additionally, Sun et al. used terahertz time-domain (THz-TDS) spectroscopy and spectral imaging technologies to explore the feasibility of the detection of insect foreign bodies in finished tea products. Using THz-TDS, significant signals differences between tea leaves and insect foreign bodies can be observed in the range of 0.3–1.0 THz. In addition, the locations and outlines of insect bodies were able to be presented *via* the THz-TDS image. The binary classification model was used on 176 samples of tea and insect mixtures to determine whether the tea contained insect foreign bodies. Three algorithms, including adaptive iteratively reweighted penalized least squares (AirPLS), were used for spectral preprocessing in order to reduce baseline drift and enhance effective spectral information. The results demonstrated that the K-nearest neighbor (KNN) and partial least squares–discrimination analysis (PLS-DA) models showed great performance after AirPLS correction. THz-TDS spectroscopy technology was capable of detecting insect foreign bodies in finished tea products; the THz-TDS imaging was able to observe the position and the profile of the insect foreign bodies and therefore provided a visual detection method for tea containing insect foreign bodies. Ultimately, these results showed that THz-TDS and imaging technology provided an effective tool for non-destructive testing of low-density and organic foreign bodies in food processing.

Aquaphotomics, another emerging analytical technology, was also employed for food analysis within this Research Topic. Aquaphotomics uses changes in specific disturbance factors (e.g., temperature, pressure, the addition of other components) to perform dynamic spectral measurement of aqueous-system samples. It further uses the chemometric method to study high-dimensional spectra corresponding to perturbation, thereby achieving quantitative and qualitative analysis of the characteristic in the aqueous-system samples. Zhang et al. used near-infrared (NIR) spectroscopy combined with aquaphotomics to study the structure of peanut allergen protein Ara h1 during the heating process. This method revealed the interaction between the water structure and Ara h1 from the perspective of the water molecules and explained the effect of temperature on the secondary structure and the hydrogen bond network of Ara h1. The results indicated that, during the heating process, the mutation temperature was about 55°C, at which point the hydrogen bond network was destroyed, free water was increased, and the content of the protein's secondary structure was changed. This study established a method for characterizing protein structural changes without labeling that is simple to operate and that has high sensitivity, and that can provide new technical ideas for the analysis of food processing.

The articles in this Research Topic emphasize and demonstrate the developments of advanced analytical techniques for modern food analysis. We hope they will inspire (young) scientists to push advances in this area even further, so that they can lead food science and nutrition research into a new era. We would like to express our great gratitude to the contributors, and our thanks to them for providing up-to-date and high-quality original contributions. We are also very grateful to the editorial team and reviewers for their support and excellent cooperation.

## Author contributions

All authors contributed to the article and approved the submitted version.

## Conflict of interest

The authors declare that the research was conducted in the absence of any commercial or financial relationships that could be construed as a potential conflict of interest.

## Publisher's note

All claims expressed in this article are solely those of the authors and do not necessarily represent those of their affiliated organizations, or those of the publisher, the editors and the reviewers. Any product that may be evaluated in this article, or claim that may be made by its manufacturer, is not guaranteed or endorsed by the publisher

